# Supplementary material for: PPE51 Is Involved in the Uptake of Disaccharides by Mycobacterium tuberculosis
Source: Cells. 2020 Mar 3;9(3):603. doi: 10.3390/cells9030603 (PMC7140425; doi:10.3390/cells9030603)
Supplement: Supplementary file 1 [file cells-09-00603-s001.zip › Korycka et al - Supplementary Materials-R1.pdf]

Supplementary Materials

PPE51 is involved in the uptake of disaccharides by *Mycobacterium tuberculosis*

Małgorzata Korycka-Machała[1], Jakub Pawełczyk[1], Paulina Borówka[2,3], Bożena Dziadek[4], Anna Brzostek[1], Malwina Kawka[4], Adrian Bekier[4], Sebastian Rykowski[1], Agnieszka Olejniczak[1], Dominik Strapagiel[2,3], Zbigniew Witczak[5] and Jarosław Dziadek[1]*

Figure S1. Purification of *M. tuberculosis* PPE51 protein

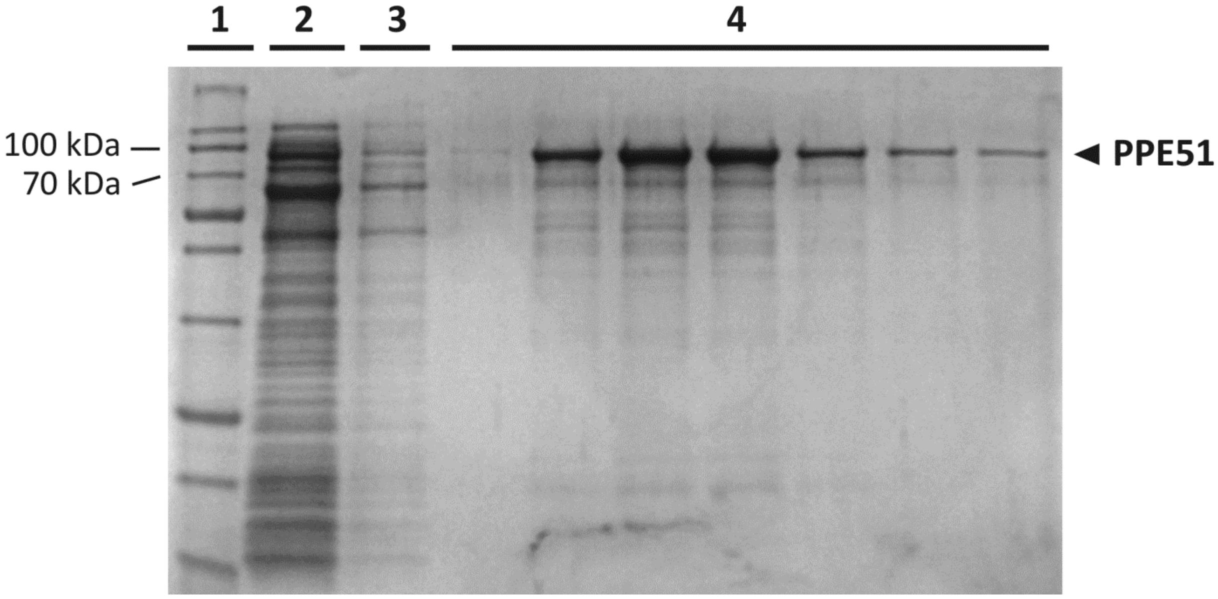

SDS-PAGE analysis. Purified protein was resolved on a 12% polyacrylamide gel followed by Instant*Blue* (Expedeon) staining. Line: 1 – molecular weight marker, 2 – flow-through, 3 – wash buffer fraction, 4 – purified PPE51 fractions.

Table S1. The thio- functionalized carbohydrate derivatives used in this study

Compound	LogP/cLogP	References
1,6-anhydro-3-deoxy-4-S-(2,3,4,6-tetra-O-acetyl-β-D-glucopyranosyl-D-glycero-*hexopyranos*-2-ulose (**6**)	LogP: -1.13<br>cLogP: 0.4407	[1,2]
1,6-anhydro-3-deoxy-4-*S*-(5-amino-1,3,4-thiadiazol-2-yl)-D-glycero-*hexopyranos*-2-ulose (**23**)	LogP: 1.37<br>cLogP: -0.535599	[3]
1,6-anhydro-3-deoxy-4-*S*-(4,5-dihydrothiazol-2-yl-D-glycero-*hexopyranos*-2-ulose (**26**)	LogP: 1.13<br>cLogP: 0.0390999	[3]

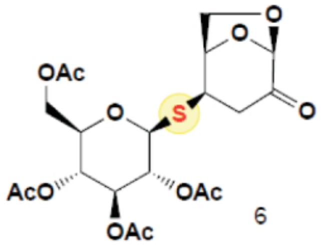

6

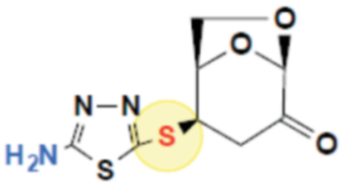

23

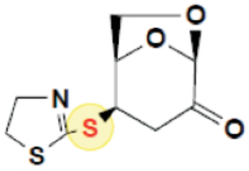

26

Table S2. Plasmids used for cloning

Plasmid	Description	Source or reference
pJET 1.2	Blunt cloning vector, Amp^R	ThermoScientific
pHIS	Expression vector, Amp^R	[4]
pMALC4e	Expression vector, Gm^R	[5]
pMV306	Mycobacterial *attB*-integrating vector, Kan^R	Med-Immune Inc.
pMK250	*ppe51* and upstream sequence 694 bp in pMV306 vector, Kan^R	This study
pMK251	*ppe51* (Ala96Ser*)* and upstream sequence 694 bp in pMV306 vector, Kan^R	This study
pMK252	*mmpL3* (Ala66Pro*)* and upstream sequence 694 bp in pMV306 vector, Kan^R	This study
pMK253	*mmpL3* (Thr83Lys) and upstream sequence 694 bp in pMV306 vector, Kan^R	This study

Table S3. Oligonucleotides used

Oligonucleotide	Sequence
**Primers used to clone genes for complementation experiments**	
RvPPE51-pr-Xba-s	5' CTCTAGATCAATCTCCGATCCGCCGTCA
RvPPE51-HindIII-r	5' CAAGCTTGCCGACCGTCGGTCAAATACTTCC
**Primers used to clone genes for protein purification**	
PPE51His-BamHI-s	5’CGGATCCGATGGATTTCGCACTGTTACCACCGG
PPE51His-HindIII-r	5' CAAGCTTACCCTGCCGCGGGTGGGT-3’
**Oligonucleotides used for CRISPR-Cas9**	
PPE51CRISPRstrong216FOR	5' GGGAATCGCCGCAGTGTTCTGGCC
PPE51CRISPRstrong216REV	5' AAACGGCCAGAACACTGCGGCGAT
PPE51CRISPRweak38FOR	5’ GGGAATATCCAGCGAATATGGCGT
PPE51CRISPRweak38REV	5’ AAACACGCCATATTCGCTGGATAT
**Oligonucleotides used for qPCR**	
qPCR*ppe51*-s	5’ CACTGTCGCTGCTGATTGAGACGG
qPCR*ppe51*-r	5’ TCAAAAGGCCTAGGTTGCTCTCGG
qPCR*ppe50*-s	5’ GCGTATGTACAGCGGTCCCGGAC
qPCR*ppe50*-r	5’ CCGCTCCAGCCATTCCACGTATG

Table S4. Mutations identified in *Mtb* T-6 resistant strains

See Excel file

Table S5. The evaluation of the sensitivity of mutants to the *thio*-glycosides T-23 and T-26

Strains	T-23<br>MIC_{90} [μM]	T-26<br>MIC_{90} [μM]
Rv	100	125
PPE51 (A96S) –T-6/2	100	125
PPE51 (A66fs) –T-6/7	125	125
PPE51 (T83K) –T-6/10	125	125
PPE51 (L95P) –T-6/22	100	125

T-23 - 1,6-anhydro-3-deoxy-4-S-(5-amino-1,3,4-thiadiazol-2-yl)-D-glycero-hexopyranos-2-ulose

T-26 - 1,6-anhydro-3-deoxy-4-S-(4,5-dihydrothiazol-2-yl-D-glycero-hexopyranos-2-ulose

A66fs - 196G-deletion, frame shift mutation

1. Witczak, Z.J.; Kaplon, P.; Dey, P.M. Thio-sugars vii. Effect of 3-deoxy-4-s-(beta-d-gluco- and beta-d-galactopyranosyl)-4-thiodisaccharides and their sulfoxides and sulfones on the viability and growth of selected murine and human tumor cell lines. *Carbohydrate research* **2003**, *338*, 11-18.
2. Witczak, Z.J.; Chhabra, R.; Chen, H.; Xie, X.Q. Thiosugars .2. A novel approach to thiodisaccharides - the synthesis of 3-deoxy-4-thiocellobiose from levoglucosenone. *Carbohydrate research* **1997**, *301*, 167-175.
3. Korycka-Machala, M.; Brzostek, A.; Dziadek, B.; Kawka, M.; Poplawski, T.; Witczak, Z.J.; Dziadek, J. Evaluation of the mycobactericidal effect of thio-functionalized carbohydrate derivatives. *Molecules* **2017**, *22*, 812-826.
4. Sheffield P, G.S., Derewenda Z. Overcomming expression and purificatio problems of rhogdi using a family of "parallel" expression vector. *Protein Expression and Purification* **1999**, *15*, 34-39.
5. Walker, I.H.; Hsieh, P.C.; Riggs, P.D. Mutations in maltose-binding protein that alter affinity and solubility properties. *Applied microbiology and biotechnology* **2010**, *88*, 187-197.
